# Examining Children and adolescent mental health trajectories during the COVID‐19 pandemic: Findings from a year of the Co‐SPACE study

**DOI:** 10.1002/jcv2.12153

**Published:** 2023-03-18

**Authors:** Carolina Guzman Holst, Lucy Bowes, Polly Waite, Simona Skripkauskaite, Adrienne Shum, Samantha Pearcey, Jasmine Raw, Praveetha Patalay, Cathy Creswell

**Affiliations:** ^1^ Department of Experimental Psychology University of Oxford Oxford UK; ^2^ Department of Psychiatry University of Oxford Oxford UK; ^3^ School of Psychology and Clinical Language Sciences University of Reading Reading UK; ^4^ Centre for Longitudinal Studies and MRC Unit for Lifelong Health and Ageing University College London London UK

**Keywords:** adolescents, children, mental health, pandemic, wellbeing

## Abstract

**Background:**

A major concern throughout the COVID‐19 pandemic has been on young people's experiences with mental health. In this study we mapped children and adolescents' mental health trajectories over 13 months of the pandemic and examine whether family, peer, and individual‐level factors were associated with trajectory membership.

**Methods:**

This study focuses on a sub‐sample from the Co‐SPACE study of 3322 children and adolescents (aged 4–16 years) for whom parents completed a survey at Time 0 and at least one follow‐up survey between March 2020 and May 2021. We used growth mixture models to examine trajectories in emotional, conduct, and hyperactivity/inattention difficulties using the Strengths and Difficulties Questionnaire and multinomial logistic regression models to estimate factors associated with individual trajectory membership.

**Results:**

The average trend in young people's mental health appeared to follow changes in national guidelines regarding the pandemic. Distinct trends in GMM models highlighting individual differences showed that a 5‐trajectory model best explained the changes in emotional problems whilst 4‐trajectory models best explained variation in hyperactivity/inattention and conduct problems. While most young people followed low stable (62%–85%) or moderate stable (28%) symptom trajectories, 14%–31% experienced very high, high stable or increasing mental health difficulties. Young people following high stable trajectories were more likely to have special educational needs and/or neurodevelopmental disorders, parents reporting higher levels of distress and parent‐child conflict, and were less likely to have at least one close friend.

**Conclusions:**

Most young people adapted well and experienced low stable symptoms, but nearly one third experienced high stable or increasing mental health difficulties. Young people with complex needs and parents with higher psychological distress were particularly vulnerable to high stable problems while those with positive peer relationships were less vulnerable. This study offers insight into potential factors that can be addressed using targeted interventions to improve the wellbeing of parents and young people in the event of future lockdowns and school closures.

## Graphical abstract

This study used data from the Co‐Space Study to examine children and adolescents' mental health trajectories over 13 months of the pandemic and whether family, peer, and individual‐level factors were associated with trajectory membership. We found that most young people adapted well and experienced low stable symptoms, but nearly one third experienced high stable or increasing mental health difficulties. Young people with complex needs and parents with higher psychological distress were particularly vulnerable to high mental health difficulties while those with positive peer relationships were less vulnerable. This study offers insight into potential factors that can be addressed using targeted interventions to improve the wellbeing of parents and young people in the event of future lockdowns and school closures.

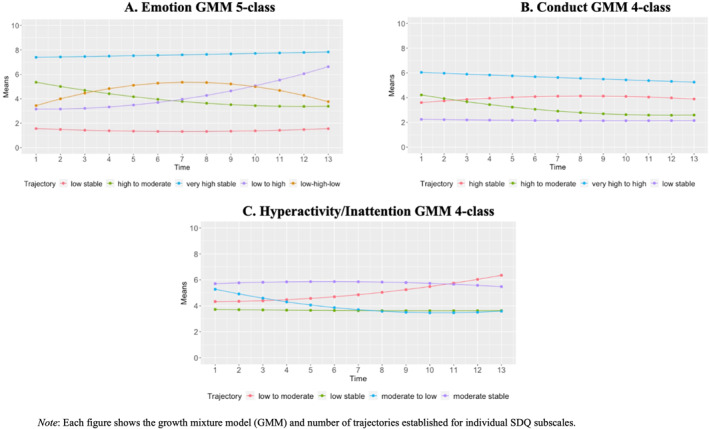

**Key points**

- This study provides insight into participating children and adolescent's mental health trajectories over the first 13 months of the COVID‐19 pandemic.
- Nearly one third of young people experienced high stable or increasing mental health difficulties. The majority followed low‐stable symptom trajectories.
- Young people with pre‐existing special educational needs or neurodevelopmental disorders were most at risk of increasing or persistent mental health problems, whilst having at least one good and supportive friend was more likely to buffer young peoples' risk of mental health problems during the pandemic.
- Policies should be in place for the immediate provision of targeted mental health and social support interventions to support the psychological wellbeing of young people identified as vulnerable to negative mental health trajectories during the COVID‐19 pandemic.

## INTRODUCTION

The COVID‐19 pandemic caused unprecedented and substantial disruption to families' lives, with a range of pressures arising for children, young people, parents, and carers. One major public health concern throughout the pandemic has been on children and young people's mental health. In England, national rates of probable mental disorders in young people increased from 12% prior to the pandemic in 2017% to 17% in 2020 and remained similar in July 2021 (NHS Digital, 2021). However, other studies reported only a 6% increase in adolescent mental health problems (Mansfield et al., 2022), while studies in specific areas, such as densely populated inner cities, reported a weighted estimated increase of 18% (Knowles et al., 2022). While these studies provide important snapshots, understanding how children and adolescent's mental health changed during the COVID‐19 pandemic can directly inform policy and practice, and bring benefits for families' wellbeing.

Several studies have looked at how young people's mental health changed during the pandemic. For example, the COVID‐19: supporting parents, adolescents, and children during epidemics (Co‐SPACE) study was set up to track the mental health and wellbeing of children and young people in the UK on a monthly basis during the pandemic. Findings showed that parental reports of general trends in children and adolescent's conduct/behavioural, emotional, and attentional difficulties changed considerably throughout the pandemic, increasing in times of national lockdown from June 2020 and February 2021, and decreasing as restrictions eased and schools reopened from February to April 2021. Studies have also shown that some children and adolescents were more vulnerable than others to the mental health effects of the pandemic. For example, a cross‐sectional study suggested that adolescents with known risk factors such as being female, experiencing food poverty or previously accessing mental health support were at greatest risk of high depression, anxiety, and low levels of wellbeing (Mansfield et al., 2021). Other studies found a higher prevalence of emotional and behavioural difficulties among primary school aged children, individuals with pre‐existing mental health diagnosis, neurodevelopmental disorders and special educational needs over the first lockdown period (Asbury et al., 2021; Cost et al., 2021; Nonweiler et al., 2020; Waite et al., 2021). However, not all young people experienced deteriorations in mental health—some adolescents experienced an improvement in their mental wellbeing during the first lockdown period in the UK (Soneson et al., 2022; Widnall et al., 2020). Indeed, a cross‐sectional study of 16,940 children and young people from the OxWell Study found that 33% of individuals self‐reported improvements in their mental health early in the pandemic (Soneson et al., 2022). It is likely that these children and those not reporting any worsening mental health symptoms had protective factors in place to offset any stress created by the pandemic. For example, several systematic reviews found that greater coping abilities, emotion regulation strategies and self‐esteem, quality of family relationships and social support, among others were important in offsetting mental health difficulties (Theberath et al., 2022; Wolf & Schmitz, 2022).

Although most studies have described overall trends in children's mental health outcomes, further evidence from the Co‐SPACE study suggests that children in the UK followed different patterns of mental health symptoms during the first 4 months of the pandemic (Raw et al., 2021). While most experienced low and stable emotional, conduct and hyperactivity/inattention problems, a significant portion also experienced increasing, high stable and decreasing symptom trajectories. To date, we only have evidence for young people following different trajectories over the first few months of the pandemic, but none to suggest whether these trajectories are sustained over a longer period, including multiple lockdowns and constant changes to daily life. Furthermore, this study also found that children and young people with elevated symptoms at the start of the study were likely to be younger, have parents with higher levels of psychological distress, have special educational needs or a neurodevelopmental disorder, or come from families with higher levels of reported family conflict compared to children with low stable symptoms. However, groups who started with elevated scores but then experienced a reduction in symptoms did not differ from groups who had increasing or stable high symptoms when they were compared to the low stable group (Raw et al., 2021). It is imperative that we built upon these findings by investigating whether these factors are associated with trajectories over a longer period, and understand what other risk and protective factors may predict specific trajectories of mental health decline or improvement.

Given the huge potential disruption to social relationships, it is also important to consider the effects of peer relationships such as friendship quality and support on mental health (Foulkes & Blakemore, 2021). For example, Co‐SPACE found that mental health difficulties were highest when social restrictions were most stringent, and schools were closed (Creswell et al., 2021), suggesting that limited social interactions could have negatively influenced children's mental health. Yet other studies with older age groups show that a significant portion of adolescents saw improvements or no changes in mental health after the first lockdown (Soneson et al., 2022; Vizard et al., 2020; Widnall et al., 2020), indicating that limited social contact (among other factors) might have a protective effect for some adolescents, most likely for those victimized or excluded by their peers. Indeed, children and young people who reported a perceived improvement in mental wellbeing also reported experiencing reduced bullying, less loneliness and exclusion compared to peers who reported no change or perceived deterioration in mental wellbeing (Soneson et al., 2022). Understanding the associations between these factors and mental health trajectories over more than a year of the pandemic can help us protect children and adolescents who are vulnerable and more at‐risk of developing negative outcomes.

For the present study, our first aim was to map children and adolescent's trajectories in mental health symptoms over 13 months of the pandemic from March 2020 to May 2021, during which there were ongoing changes in restrictions to daily activities and lifestyles. We used parent‐reported emotional, conduct and attentional/hyperactivity difficulties sub‐scales from the Strengths and Difficulties Questionnaire (SDQ). Our second aim was to investigate if different trajectories in children and young people's mental health over time were associated with (i) child demographics (age, gender, ethnicity), (ii) household income (living in poverty or not), (iii) presence of pre‐existing conditions (physical, mental health, special educational needs and neurodevelopmental disorders), (iv) parental distress, (v) parent‐child relationship, and (vi) friendship quality and support. Our goal was to understand if certain factors were associated with children and young people's difficulties during the pandemic to inform practice in case of future extended lockdown periods, and to identify and protect more vulnerable children and young people.

## METHODS

### Design

The Co‐SPACE study is an online longitudinal study comprised of a convenience sample of UK parents and carers (hereafter known as parents). The research protocol of the main study is available via the Open Science Framework (OSF; http://osf.io/8zx2y/), where more details on the eligibility, recruitment, procedure, and participants can be found. The pre‐registered protocol of the current study can be found at: https://osf.io/ajndw.

### Participants

The current study focussed on a sub‐sample of children and young people (*N* = 3322) for whom parents completed a survey at ‘Time 0’ between 30th March and 29th April 2020 and then completed at least one follow‐up survey between 1st May 2020 to 31st May 2021. Only those who completed at least 3/5 items for each subscale of the Strengths and Difficulties Questionnaire (SDQ; Goodman, 1997, Goodman, 2001) at Time 0 and follow‐ups were included in the analysis. Participants with missing data on predictor variables at Time 0 were not excluded from the analysis to avoid biases and loss of power (see Appendix S1 for note on missing data).

### Procedure

A convenience sample of parents were invited to take part in an online survey using Qualtrics software from late March 2020. Parents with multiple children were asked to complete a survey for just one child. After completing a survey at Time 0 between March and April 2020 (first month of lockdown in the UK), parents were invited to complete monthly follow‐up surveys until May 2021. Informed consent was obtained from parents. From December 2020 participants had an opportunity to enter a prize draw every month to win one £50 voucher. Ethical approval for the study was provided by the University of Oxford Medical Sciences Division Ethics Committee (reference R69060).

### Measures

Our main outcome measure was assessed monthly over the first 13 months of the pandemic and other measures were assessed only at Time 0 (see Table 1).

**TABLE 1:** Description of the measures used in the study.

	Measures	Rating scale	Coding
Main outcomes			
Child mental health	Parents completed the parent‐report version of the strengths and difficulties questionnaire (SDQ;Goodman, 2001) which is a widely used measure of mental health in children. We used the subscales for emotional, conduct and hyperactivity/inattention difficulties.	Each subscale is comprised of 5 items and scored using a 3‐pointlikert scale ranging from 0 (“*not at all*”) to 2 (“*certainly true*”)	Subscale sums were treated as continuous variables. Missing items were pro‐rated using SDQ guidance (www.sqdinfo.org).
Baseline variables			
Child's pre‐existing mental health	Parents reported on whether their child had any pre‐existing diagnosed mental health disorders (including depression, anxiety or other)		All variables were coded as binary
Child's pre‐existing special educational needs or neurodevelopmental disorders (SEN/ND)	Parents reported on whether their child had any pre‐existing special educational needs or neurodevelopmental disorders (including autism spectrum and attention deficit/hyperactivity disorders)		All variables were coded as binary
Child's pre‐existing medical condition	Parents reported on whether their child had any pre‐existing medical condition including chronic illnesses (e.g., diabetes, cancer).		All variables were coded as binary
Child's demographic information	Parents were asked questions about their child's demographic background including age, gender, ethnicity, and total household income.		Age (continuous), gender (binary—male or female), ethnicity (binary ‐ white or other). Total household income was coded into more than £16k per year and less than £16k per year as his reflects an income below 60% of the median income in the United Kingdom. Approximately 6% of reporters chose not to specify their household income (e.g., “I*prefer not to say*”). We treated these values as missing and examined different income thresholds using sensitivity analysis (see Appendix S2).
Parent‐child relationship	Measures were obtained using responses to the following statements: ‘*My child and I have a warm, close relationship*’ (parental warmth) and ‘*My child and I argue a lot*’ (parent‐child conflict)	Each statement is rated on a 4‐point likert scale ranging from 0 (“*Not at all”*) to 3 (“*Completely”*), in addition to an option of “*Not applicable*”.	These two variables were treated as separate, continuous variables—parental warmth and parent‐child conflict.
Parent psychological distress	Symptoms of psychological distress in parents at baseline were assessed using a subset of 9‐items (McElroy et al., 2020) from the depression anxiety stress scales (DASS‐21; Antony et al., 1998)	Items were rated on a 4‐point scale: 0 (“*Did not apply to me at all*”) to 3 (“*Applied to me very much, or most of the time*”)	An overall score was obtained by summing the scores for each item with a total maximum score of 27.Responses were treated as continuous.
Friendship quality and support	Parents were asked whether their child had “*at least one friend that they can turn to for support.”*	This was rated on a 4‐point scale: 0 (“*not at all*”), 1 (“*a bit”),* 2 (“*a lot*”) and 3 (“*completely*”).	Responses were dichotomized by “*not at all*” and “*a bit*” coded as 0 and “*a lot*” and “*completely*” coded as 1

### Statistical analysis

We used descriptive statistics to analyse participant demographics and characteristics at Time 0 using *R* (R Core Team., 2018; v.4.1.2). For our main analysis we used growth mixture models|growth mixture model (GMM) to identify different trajectories (Jung & Wickrama, 2008) in Mplus version 8.7 (Muthén & Muthén, 2017). The three sub‐scales of the SDQ: emotional symptoms, conduct problems and hyperactivity/inattention were treated as the dependent variables and modelled separately. A time variable was devised based on the month of survey completion (e.g. a survey completed between the 1st and 30th of June was assigned to June). The months were assigned a numerical value from ‘0’ for March/April 2020 to ‘13’ for May 2021. If participants had multiple entries per calendar month, then only the first entry was used in the analysis. Predictors were derived from Time 0. Missing data were addressed using full information maximum likelihood estimation (FIML). This method takes into account any of the missingness in the data without discarding any information to produce unbiased estimates. This method tends to outperform other methods such as multiple imputation when missingness levels are high (Xiao & Bulut, 2020). Trajectory names were based on the 4‐categorisation band for the parent‐rated SDQ (close to average = low, slightly raised = moderate, high and very high, see www.sqdinfo.org).

We first modelled separate latent growth curves for each subscale to determine whether linear or quadratic polynomial functions best fit the data. Estimating quadratic growth, compared to linear growth, improved the fit for all subscales, therefore quadratic functions were employed in all subsequent models. We fitted a series of models beginning with one single growth curve trajectory (our overall mean trajectory) and successively added latent classes until reaching an optimal number of groups. Following this approach, we compared a latent class growth analysis model with all within‐class variance for intercept, slope and quadratic factor fixed to 0 to a constrained GMM model with within‐class variances for the intercept equal but freely estimated and the slope and quadratic factors fixed to 0. The GMM model revealed a better fit based on the statistical indicators. To determine a good statistical fit, we used Comparative Fit Measure (CFI) and Root Mean Square Error of Approximation (RMSEA) with the following cut‐off values: CFI ≥0.95, RMSEA <0.05 (Hu & Bentler, 1999). Bayesian information Criterion, Entropy Index and the Vuong‐Lo‐Mendell‐Rubin Likelihood Ratio Test (VLMR‐LRT; Lo et al., 2001) were used to determine the optimal number of groups. Specifically, we used a low Bayesian information Criterion value, higher entropy value (near 1.0) and a significant VLMR‐LRT *p*‐value comparing *k* and k‐1 class to guide our decision. Other considerations included successful convergence and ease of interpretability. For all model indices and comparisons see Appendix S3. To avoid problems related to nonconvergence and local maxima we increased the number of random sets, optimizations and start iterations (see Appendix S6 for Mplus code).

Lastly, to answer our second question, we regressed group membership on to different family, peer and individual variables using multinomial logistic regression for each SDQ sub‐scale separately. As most entropy values were <0.80, class probability weights were included in the regression models to account for the lower neatness of classification. We report results using low stable and high stable groups as references to provide more insight into our data. We set the alpha level to 0.05 and report odds ratios (OR) with respective 95% confidence intervals (CI).

## RESULTS

### Participant characteristics

Table 2 shows demographic information and characteristics at Time 0 for respondents and their children.

**TABLE 2:** Sample demographics and baseline characteristics.

Baseline characteristics	Frequency (%)
Child age M(SD)	9.25 (3.46)
Children (4–10 years)	2176 (65.5)
Adolescents (11–16 years)	1146 (34.5)
Child gender	
Female	1592 (47.9)
Male	1708 (51.4)
Prefer not to say or missing	22 (0.7)
Child ethnicity	
White (British, Irish or other)	3065 (92.3)
Non‐white	241 (7.3)
Prefer not to say or missing	16 (0.5)
Pre‐existing child mental health problems	
No mental health problems	3125 (94.1)
Mental health problems	197 (5.9)
Child health vulnerability	
No health vulnerability	3036 (91.4)
Health vulnerability	286 (8.6)
Child SEN/ND	
No SEN/ND	2736 (82.4)
SEN/ND	586 (17.6)
Parent psychological distress M(SD)	5.21 (4.46)
Prefer not to say or missing	6 (0.2)
Family income	
£16,000–£29,000 a year	360 (10.8)
£30,000–£59,000 a year	986 (29.7)
£60,000 ‐ £89,000 a year	751 (22.6)
£90,000‐£120,000 a year	419 (12.6)
More than £120,000 a year	406 (12.2)
< £16,000 a year (<£310 a week)	171 (5.1)
≥ £16,000 a year (>£310 a week)	2922 (88.0)
Prefer not to say^a^	229 (6.9)
Friendship quality and support	
Has no good friends	1552 (46.7)
Has at least one good friend	1768 (53.3)
Prefer not to say or missing	2 (0.1)
Parent child conflict M(SD)	0.85 (0.66)
Prefer not to say or missing	11 (0.3)
Parent child warmth M(SD)	2.70 (0.55)
Prefer not to say or missing	8 (0.2)

*Note*: SEN/ND = special educational needs or neurodevelopmental disorders.

^a^ All 229 “prefer not to say” answers were considered missing.

### Changes in children and adolescent's mental health over 1 year of the pandemic

**FIGURE 1:**
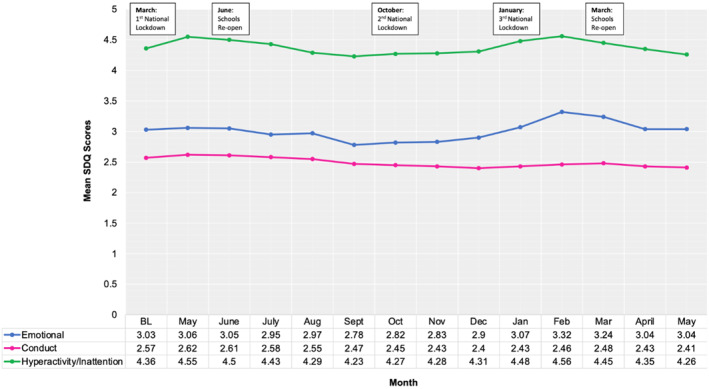
Overall trends in Children and Adolescent's Mental Health (2020–2021). BL, Baseline or time 0 (March/April 2020); SDQ, strengths and difficulties questionnaire.

Based on visual inspection (Figure 1), the average trend in participating young people's emotional, conduct and hyperactivity/inattention difficulties appeared to follow changes in government restrictions and national guidelines in the UK (Institute for Government, 2021). Most mental health difficulties increased and peaked after the national lockdowns were announced and saw a decline over periods when restrictions eased, and schools re‐opened. For a full narrative synthesis see Appendix S4.

### Trajectories of emotional, conduct and hyperactivity difficulties across the pandemic

**FIGURE 2:**
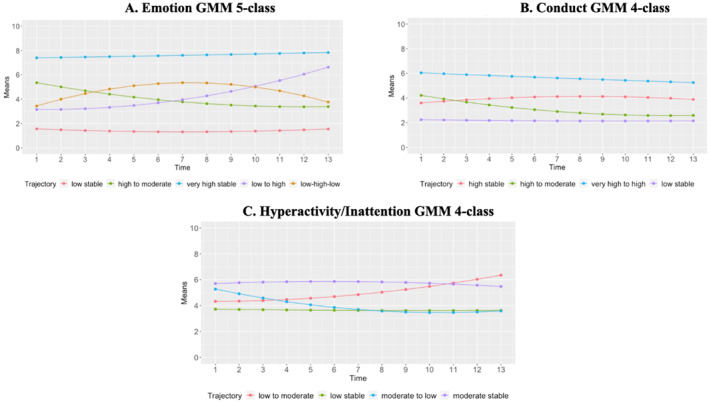
Children and Adolescents' Mental Health Trajectories over 13 months of the pandemic. Each figure shows the growth mixture models|growth mixture model (GMM) and number of trajectories established for individual Strengths and Difficulties Questionnaire (SDQ) subscales.

Trajectories were examined using growth mixture models (GMM, see Table 3). For emotional difficulties, we chose a five‐trajectory model. This model identified a low stable group (*n* = 2067), a high to moderate group (*n* = 452), a low to high group (*n* = 211), a low‐high‐low group (*n* = 234) and a very high stable group (*n* = 358; see Figure 2A). For conduct difficulties, we chose a four‐trajectory model. This model identified a low stable group (*n* = 2795), a high stable group (*n* = 291) a very high to high group (*n* = 92) and a high to moderate group (*n* = 144; see Figure 2B). For hyperactivity/inattention difficulties, we chose a four‐trajectory model. This model identified a low stable group (*n* = 2137), a moderate stable group (*n* = 931), a low to moderate group (*n* = 160), and a moderate to low group (*n* = 94); see Figure 2C). Notably, the entropy index for this model was very low (value = 0.59) suggesting problems with classification. We explored this further by performing a sub‐group analysis for hyperactivity/inattention difficulties based on age group (children or adolescents) and found improvement for classification in adolescent trajectories but not in child trajectories (see Appendix S2 for subgroup‐analysis).

**TABLE 3:** Results for growth mixture models|growth mixture model (GMM) model group classifications.

SDQ subscale	Trajectory	N (%)
Emotion		
Low stable (low)	1	2067 (62.22)
High to moderate (decreasing)	2	452 (13.61)
Very high stable (high)	3	358 (10.78)
Low to high (increasing)	4	211 (6.35)
Low‐high‐low (both increasing and decreasing)	5	234 (7.04)
Conduct		
High stable (high)	1	291 (8.76)
High to moderate (decreasing)	2	144 (4.34)
Very high to high (decreasing)	3	92 (2.76)
Low stable (low)	4	2795 (84.14)
Hyperactivity/Inattention		
Low to moderate (increasing)	1	160 (4.82)
Low stable (low)	2	2137 (64.33)
Moderate to low (decreasing)	3	94 (2.83)
Moderate stable (moderate)	4	931 (28.03)

### PREDICTORS OF TRAJECTORY MEMBERSHIP

**FIGURE 3:**
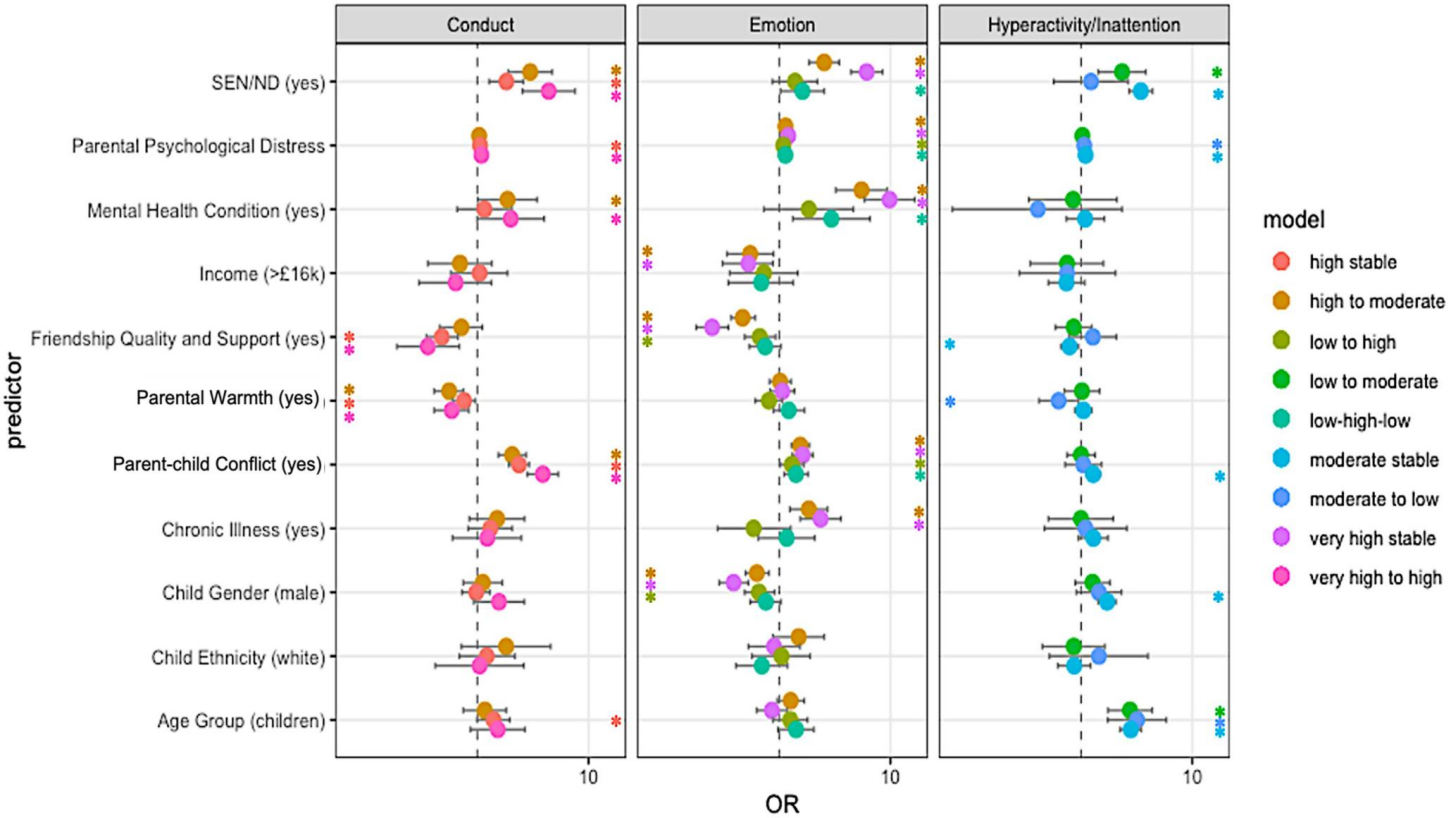
Factors associated with trajectory membership (Odds Ratio and 95% confidence interval) ‐ using low stable groups as reference. * Statistically significant odds ratios (OR) at 0.05.

**FIGURE 4:**
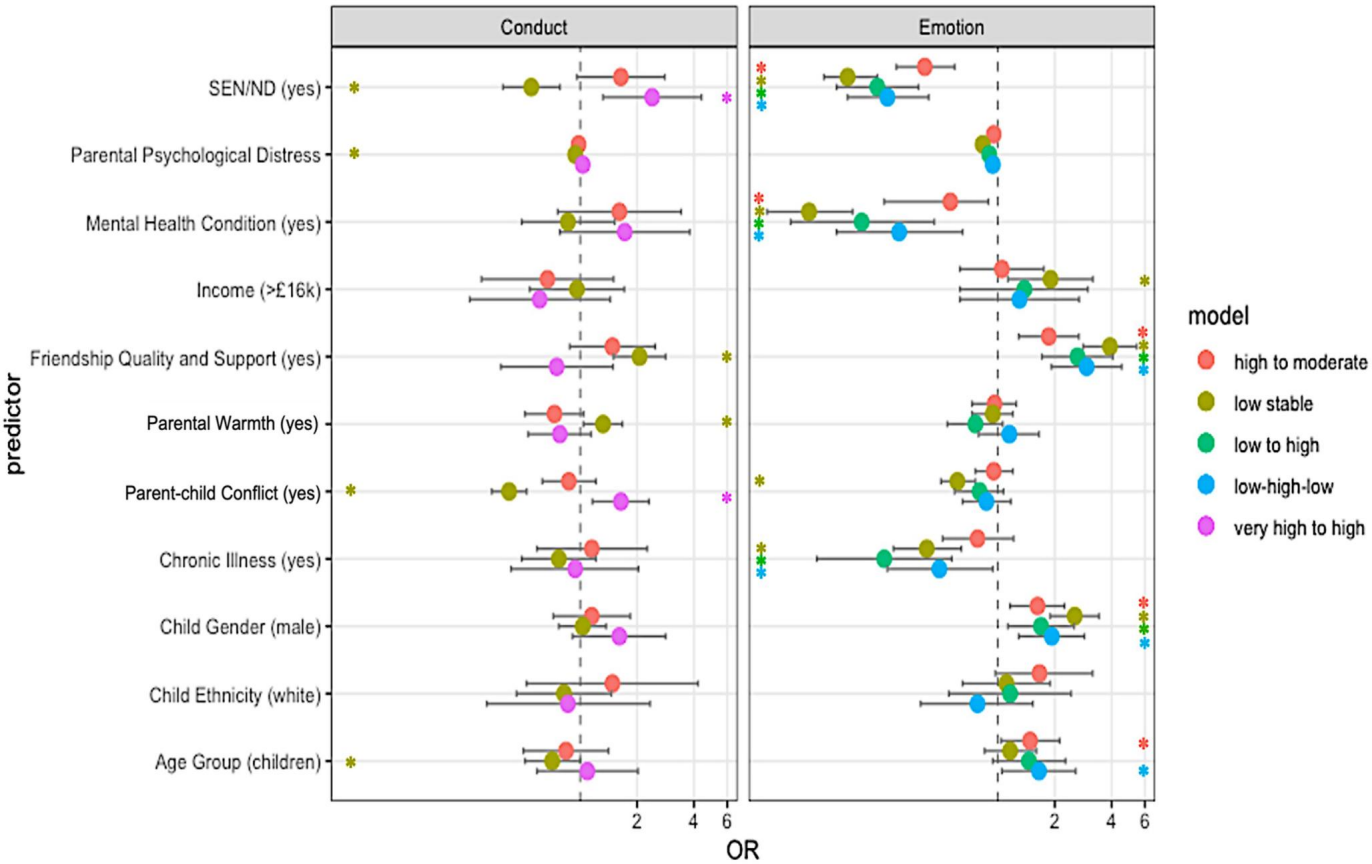
Factors associated with trajectory membership (Odds Ratio and 95% confidence interval) ‐ using high stable groups as reference. * Statistically significant odds ratios (OR) at 0.05.

We report only statistically significant OR with 95% confidence intervals for multinomial analyses using low stable (Figure 3) and high stable groups (Figure 4) as reference. For the hyperactivity/inattention subscale we only report on the low stable group as a reference as there is no high stable group. For all model results see Appendix S5.

### Emotional difficulties

Compared to the low stable reference group, children and adolescents in all other groups (very high stable, low to high, high to moderate and low‐high‐low) were more likely to have parents who reported higher levels of psychological distress and higher levels of parent‐child conflict. Apart from those in the low to high group, children and adolescents in all other groups (particularly in the very high stable group) were more likely to have a SEN/ND or a pre‐existing mental health condition compared to the low stable group. Except for the low‐high‐low trajectory group, children and adolescents in all other groups were also less likely to be male or have at least one friend they could turn to for support compared to the low stable reference group. Children and adolescents in the very high stable trajectories and high to moderate trajectories were also less likely to come from a family with a household income above £16k, more likely to have a chronic illness or mental health condition than those in the low stable reference group (see Appendix S5, Table S8).

Compared to the high stable reference group, children and adolescents in all other groups were less likely to have a pre‐existing mental health condition, have a SEN/ND, or to have a parent who reported higher levels of psychological distress at Time 0. They were more likely to be male and to have at least one friend they could turn to for support. Except for children and adolescents in the high to moderate symptom (decreasing) trajectory group, those in all other groups were less likely to have a chronic illness. Compared to the high stable reference group, young people in the high to moderate and low‐high‐low (i.e., involving decreasing symptom trajectories) were more likely to be younger. Those in the low stable groups were more likely to come from a family with household incomes above £16k and less likely to come from a family where parents reported higher levels of conflict (see Appendix S5, Table S9).

### Conduct difficulties

Compared to the low stable reference group, children and adolescents in all other groups, were more likely to have a SEN/ND and come from a family where parents reported higher parent‐child conflict. They were also less likely to come from a family where parents reported higher levels of warmth. Compared to the low stable reference group, children and adolescents in the high stable group were more likely to be children (than adolescents), more likely to have a parent who reported higher levels of psychological distress and less likely to have at least one close and supportive friend. Compared to the low stable reference group, children and adolescents in the very high to high (decreasing) group, were more likely to have a parent who reported higher level of psychological distress, more likely to have a pre‐existing mental health condition and less likely to have at least one close and supportive friend. Compared to the low stable reference group, children and adolescents in the high to moderate (decreasing) group were more likely to have a pre‐existing mental health condition (see Appendix S5, Table S10).

Compared to the high stable reference group, children and adolescents in the low stable group were less likely to be younger, less likely to have a parent who reported a higher level of psychological distress and parent‐child conflict, less likely to have a SEN/ND and more likely to have a close and supportive friend and come from a family where the parent reported higher levels of warmth. Compared to the high stable reference group, children and adolescents in the very high to high group (decreasing) were more likely to come from a family where the parent reported higher levels of parent‐child conflict and were more likely to have a SEN/ND (see Appendix S5, Table S11).

### Hyperactivity/inattention difficulties

Compared to the low stable reference group, young people in all other groups were more likely to be children than adolescents. Compared to the low stable group, children and adolescents in the moderate stable group were more likely to be male, more likely to have a parent who reported higher level of psychological distress and higher levels of conflict, less likely to have at least one close friend they could turn to for support, and more likely to have a SEN/ND. Compared to the low stable reference group, children and adolescents in the moderate to low (decreasing) group, were more likely to have a parent who reported higher levels of psychological distress and high levels of warmth. Compared to the low stable reference group, young people in the low to moderate (increasing) group were also more likely to have a SEN/ND (see Appendix S5, Table S12).

## DISCUSSION

The aim of this study was to examine parent‐reported children and young people's mental health trajectories and their association with multiple potential risk and protective factors over 13 months of the COVID‐19 pandemic, from March 2020 to May 2021. The overall mean change in symptoms observed during the pandemic highlighted important patterns that were consistent with the drastic changes that occurred in the daily lives of young people. Whilst informative, overall trends might conceal individual differences or heterogeneity seen in symptoms. Indeed, GMM models showed that while most children followed low stable symptom trajectories over time (62%–84%), a significant portion also followed very high and high stable symptom trajectories (8%–11%) and a smaller group followed increasing symptom trajectories (4%–6%). These findings highlight how most children in our sample were resilient and adapted well to the different challenges imposed during the first year of the pandemic. Consistent with previous research that found that some young people benefitted from staying home (Asbury et al., 2021; Soneson et al., 2022; Widnall et al., 2020), some young people in our sample also followed decreasing symptom trajectories (2%–13%). Overall, these findings are in line with similar trajectory patterns identified in the first 4 months of the pandemic (Raw et al., 2021), yet unsurprisingly we found that more trajectories emerged over time. Notably, our findings are also consistent with mental health trajectories identified pre‐pandemic. For example, a systematic review found that studies typically report anywhere between 4‐6 trajectories that are characterized by low levels, moderate levels, increasing or decreasing levels and high levels of mental health difficulties (Shore et al., 2018). While our trajectories only reflect changes in mental health that occurred during the pandemic, we cannot rule out the possibility that they were a continuation of pre‐existing patterns. Unfortunately, we cannot draw these conclusions without pre‐pandemic data.

This study is unique in its ability to examine associations between different trajectories in young people's mental health throughout the pandemic and a range of known risk and protective factors. When compared to low stable symptom groups, high stable trajectories across all symptom subtypes were consistently more likely to be associated with young people having special educational needs or a neurodevelopmental disorder, high levels of parental psychological distress and parent‐child conflict, and a lower likelihood of having at least one close and supportive friend. Pre‐existing mental health was more likely to be associated with very high stable symptom groups when compared to low stable groups.

These findings are consistent with a large body of literature showing that having special educational needs and/or a neurodevelopmental disorder puts children and adolescents at risk of worsening or chronic mental health problems (Emerson & Hatton, 2007), including in pandemic times (Asbury et al., 2021; Waite et al., 2021). In the event of future pandemics, these young people are likely to benefit from targeted mental health and social support.

Our findings are also consistent with previous findings showing that high levels of family conflict and parental distress can increase the risk of child mental health problems (Cummings et al., 2015; Vostanis et al., 2006). In the context of the COVID‐19 pandemic it is likely that family stress increased as a result of working, home‐schooling, and juggling multiple responsibilities and uncertainties. This is particularly concerning for children and adolescents' mental health as parental psychological distress increased as the pandemic progressed (Racine et al., 2021; Wright et al., 2021; Skripkauskaite et al., in prep). Indeed, previous Co‐SPACE reports found that an increasing number of parents were struggling to meet the needs of their child and their work, and three‐quarters of parents reported wanting extra support (Shum et al., 2021). Moving forward it is imperative that parents have ready access to resources to support their children and cope with their own mental health in the face of another national crisis.

This study also showed that having at least one close and supportive friend protects children from following high stable or chronic mental health trajectories. Even children and adolescents in the low‐high‐low group, who experienced increased emotional difficulties at the start of the pandemic but were able to bounce back, were more likely to have supportive peer relationships when compared to a high stable reference group. This is consistent with other studies showing that close friendships can enhance resilience and improve mental health outcomes in young people (Bagwell et al., 2005; Demir et al., 2007). Indeed, limited social support has been associated with an increased risk of loneliness (Matthews et al., 2019), which in turn has been associated with an increased risk for depression and anxiety both in the short and long term (Loades et al., 2020). It is likely that social distancing and long periods of isolation were key catalysts for mental health difficulties in young people, particularly for those lacking support to begin with. Notably, a striking 47% of parents reported that their child did not have or hardly had at least one close and supportive friend at the start of the pandemic. Our findings highlight the importance of social support as a protective factor for mental health during times of uncertainty, isolation and change for young people, and support the need to revise lockdown regulations concerning social distancing for young people in the event of future pandemics.

Based on our findings, we were not able to identify why some children show improvements in symptoms throughout the pandemic. Decreasing symptom trajectories could not be distinguished from low stable or high stable groups across the different symptom subscales. This means that when compared to a reference group, the same factors that were associated with other groups, were associated with decreasing symptom trajectories. These results mirror a previous study examining children's mental health trajectories over the first 4 months of the pandemic (Raw et al., 2021), and potentially indicate that factors such as parental distress, parental warmth, having special educational needs or neurodevelopmental disorders were related to initial symptom levels (when the first national lockdown begun), as most decreasing symptom trajectories began with very high or high levels and only decreased slightly over time. Similarly, children and adolescents who started low and followed increasing symptom trajectories could not be evidently distinguished from low stable trajectory groups. It is also likely that the factors we examined do not capture all potential protective effects such as vaccine availability, wider family being available and access to support, among others, that should be looked at in future research.

Demographic characteristics were mostly associated with emotional and hyperactivity/inattention difficulties rather than conduct difficulties. Specifically, being young, female and from a household earning an income lower than 16k per year was associated with high symptom trajectories, whilst being adolescent, male, and from a household earning more than 16k a year, was associated with low symptom trajectories. These demographic trends are in line with other findings in adult populations during the pandemic (Fancourt et al., 2021) suggesting these might be universal vulnerability indicators. Further research with representative samples is needed to understand specific vulnerabilities in females and individuals coming from low‐income backgrounds and whether additional family support could safeguard them in the event of future pandemics.

### Limitations

Our findings should be understood in the context of some limitations. First and foremost, the lack of pre‐pandemic data meant we were not able to comment on how mental health may have changed as a result of the onset of the pandemic. Second, it is important to highlight that this study population was not a representative sample as it was a convenience or opportunistic sample biased towards middle and high‐income families from White British backgrounds so we cannot draw conclusions about population prevalence. Given we found differences among trajectories based on household income, and knowing that mental health symptoms are prevalent in low‐income households, it is likely that our results are an under‐estimation of true values. Furthermore, we do not know how many people chose not to participate in the study, but it is likely that our recruitment approach attracted participants from specific backgrounds, particularly those with internet access (Pierce et al., 2020).

In terms of the measures used in this study, we did not validate the presence of pre‐existing mental health disorders using clinical diagnostic manuals. Therefore, our finding that pre‐existing mental health problems was only associated with emotional difficulties might only reflect the nature of the question, as parents were asked if their child had a diagnosis for “anxiety, depression or other mental health problems.” Similarly, although we used the SDQ as a widely validated proxy for mental health difficulties or symptoms, it was not validated with other diagnostic measures or other reporters (e.g., self‐report or teacher reports). This is important as parents may not be fully aware of adolescents' personal lives and emotional difficulties. For example, concerning the friendship question, it is possible that parents may have found it hard to interpret what it means for their child to have a “at least one friend they can turn to for support” or be unaware of supportive friendships made online or through gaming. In the peer relationship literature (particularly social networks) we know there are distinctions between informal social groups and supportive or reciprocated friendships (Bollmer et al., 2005; Waldrip et al., 2008), and that peer and other informants such as teachers tend to have good reliability in reporting these friendships, particularly for primary school aged children (Gest, 2006). In this specific context it is possible parents were more aware of quality and supportive friendships (particularly for younger children) than usual, as they were spending prolonged periods of time with their children at home. Whilst parental reports may not be the most accurate at reporting peer relationships, they have been used to assess quality friendships in the past (Sakyi et al., 2015), including the widely used parent reported version of the SDQ peer‐relationships subscale. Another limitation regarding parental reports is that we did not control for parental mental health or other factors when deriving trajectories, thus it is possible that parents' perceptions of their child's mental health might be influenced by their own mental health (Najman et al., 2000; for review see De Los Reyes & Kazdin, 2005). Other Co‐Space reports looking at changes in parental mental health symptoms over 10 months of the pandemic found trajectories for parental distress, anxiety and depression that differed to the ones reported in this study (Skripkauskaite et al., 2022), suggesting that there might be a distinction between parental reports of their own versus their child's problems. Despite its limitations, the SDQ parental report has been widely used in general population samples and shown to have satisfactory agreement with child reports (Becker et al., 2004; Goodman, 2001; Van Roy et al., 2010; Van Widenfelt et al., 2003). Notably, when there is limited agreement, children and adolescents tend to report more problems than parents, meaning that problems could have been underestimated in our study.

In terms of missing data, it is important to acknowledge that there was a substantial amount of missing data as parents did not fill out surveys each month. Although we used FIML, a widely implemented and recommended method to deal with large amounts of missing data, it is possible that parameter estimates derived from the likelihood function might have been biased due to the volume of missing data, particularly in later timepoints. Missing data gradually increased throughout the study (see Figure S1); it increased steeply after the first lockdown when restrictions were lifted and decreased slightly after the summer months leading up to the second national lockdown before increasing once restrictions lifted. Indeed, it is possible that our sample could be biased towards a larger number of parents reporting negative outcomes during lockdowns or increasing frustrations as the pandemic progressed into 2021. To the best of our knowledge, only a few factors influenced attrition (see Appendix S1, Table S1). Specifically, younger, unemployed, and more distressed parents were more likely to have missing data at months 6 and 12 of the study. Although 46.8% (*n* = 1558) of our sample completed at least 6 time point surveys (including the Time 0 survey), we cannot know whether our results would be generalisable to those who only completed the survey once or twice.

In terms of our covariate analysis there may be other pandemic and non‐pandemic factors influencing trajectories over and above those included in this study. Furthermore, we only examined covariates at Time 0 and thus do not consider any changes to these factors that might have occurred throughout the pandemic. Lastly, for pragmatic reasons we included two single‐item questions to assess for parent‐child conflict and parental warmth. We suggest interpreting these findings with caution until more reliable assessments can be made.

## CONCLUSION

This study provides insight into parent‐reported children and adolescent's mental health trajectories during the COVID‐19 pandemic. Most young people experienced low stable trajectories, suggesting they adapted to major changes to everyday life. However, nearly one third experienced high stable or increasing mental health difficulties over 13 months of the pandemic. We examined the association between several individual and family factors with each trajectory group, however only four factors—having a special educational need and/or neurodevelopmental disorder, the absence of having at least one close and supportive friend, parent psychological distress and parent‐child conflict—were consistent across symptom trajectories when compared to both low and high stable groups. Our findings suggest that young people with pre‐existing special educational needs or neurodevelopmental disorders were most at risk of increasing or chronic mental health difficulties, whilst having at least one good friend to turn to for support was more likely to buffer young peoples' risk of increasing or persistent mental health difficulties. These findings underscore the need to promote social support and positive peer relationships in existing interventions and enhance targeted interventions to support young people with complex needs. Indeed, with school closures and social distancing, many young people were cut off from some of their main sources of social support, thus re‐evaluating the consequences of these measures and seeking alternatives should be a priority for future national lockdown policy. Similarly, our findings suggest that parents bore a huge amount of pressure throughout the pandemic, and those who endorsed elevated levels of psychological distress reported more negative trajectories of children's mental health. This finding highlights the importance of prioritising and improving access to psychological support for parents and increasing awareness of existing resources. This study offers a unique perspective on how known risk and protective factors were associated with young people's mental health trajectories over a period of prolonged disruption to daily life. Whilst our findings show that many young people maintained good mental health, they also help identify vulnerable young people with implications for how we can best support them in the event of another unprecedented crisis.

## AUTHOR CONTRIBUTIONS

**Carolina Guzman Holst**: Conceptualization; Formal analysis; Investigation; Methodology; Visualization; Writing – original draft; Writing – review & editing. **Lucy Bowes**: Conceptualization; Investigation; Methodology; Supervision; Writing – review & editing. **Polly Waite**: Conceptualization; Funding acquisition; Investigation; Methodology; Project administration; Supervision; Writing – review & editing. **Simona Skripkauskaite**: Conceptualization; Data curation; Investigation; Methodology; Supervision; Writing – review & editing. **Adrienne Shum**: Data curation; Writing – review & editing. **Samantha Pearcey**: Data curation; Writing – review & editing. **Jasmine Raw**: Investigation; Methodology; Writing – review & editing. **Praveetha Patalay**: Investigation; Methodology; Writing – review & editing. **Cathy Creswell**: Conceptualization; Funding acquisition; Investigation; Methodology; Project administration; Supervision; Writing – review & editing.

## CONFLICT OF INTEREST STATEMENT

Carolina Guzman Holst, Lucy Bowes, Polly Waite, Simona Skripkauskaite, Adrienne Shum, Samantha Pearcey, Jasmine Raw, Praveetha Patalay, Cathy Creswell, report no conflicts of interest.

## ETHICAL CONSIDERATIONS

Ethical approval for the study was provided by the University of Oxford Medical Sciences Division Ethics Committee (reference R69060).

## Supporting information

Supplementary Information S1

## Data Availability

The data that support the findings of this study are available from the corresponding author upon reasonable request.
