# Supplementary material for: Examining Children and adolescent mental health trajectories during the COVID‐19 pandemic: Findings from a year of the Co‐SPACE study
Source: JCPP Adv. 2023 Mar 18;3(2):e12153. doi: 10.1002/jcv2.12153 (PMC10519733; doi:10.1002/jcv2.12153)
Supplement: Supplementary file 1 — Supplementary Information S1 [file JCV2-3-e12153-s001.docx]

**Appendix S1: Missing Data and Attrition**

1. Note on missing data and attrition
2. Table S1. Mean comparisons between values of responders (Not missing) and non-responders (Missing) on the SDQ scores at 6 and 12 month follow-up
3. Figure S1. Missing data patterns across SDQ subscales for each time point
4. Figure S2. Frequency of surveys with all SDQ items completed by a parent

**Appendix S2: Subgroup and Sensitivity Analysis**

1. Child and Adolescent Models of Hyperactivity/Inattention Problems

Table S2. LCGA Hyperactivity/Inattention 1-Class Model Fit Indices

Table S3. GMM Hyperactivity/Inattention 1-Class to 5-Class Model Fit Indices

1. Income Sensitivity Analysis

Table S4. Sensitivity Analysis Income

**Appendix S3: LCGA and GMM Model Indices**

1. Table S5. Model Fit Indices LGCA
2. Table S6. Results for LCGA (all variances fixed to 0)
3. Table S7. Results for GMM (within-class variances s-q fixed to 0)

**Appendix S4: Descriptive Results from Overall Mental Health Trends**

**Appendix S5: Multinomial Regression Analysis**

1. Table S8. MNRA Emotion – Low Stable Reference
2. Table S9. MNRA Emotion – High Stable Reference
3. Table S10. MNRA Conduct – Low Stable Reference
4. Table S11. MNRA Conduct – High Stable Reference
5. Table S12. MNRA Hyperactivity – Low Stable Reference

**Appendix S6: Code Syntax**

1. Syntax Mplus LGCA
2. Syntax Mplus GMM (within-class variances fixed to 0)
3. Syntax R Multivariate Analysis

**Appendix S1: Missing Data and Attrition**

Attrition was difficult to determine because respondents were allowed to complete surveys at any follow-up time point. Figure 1 shows average missing data patterns for the SDQ subscales for our sample of 3322 participants who completed a survey at Time 0 in March or April and at least one other survey at another time point. Figure 2 shows that most parents in our sample completed between 2-4 surveys including the survey at Time 0, and 185 parents completed SDQ items in a survey at all time points. To assess differences in missing data relevant to this study, we compared mean SDQ subscale scores between respondents and non-responder characteristics at the 6-month and 12-month follow-up using non-parametric tests to account for the distribution of data (Table 1). Older respondents, and those with higher scores on the DASS-9 (assessing parental psychological distress) were significantly more likely to have missing data on their child’s SDQ scores at both follow-ups. Respondents working a full-time job were significantly more likely to have missing data than those unemployed/ homemakers and employed in other ways (e.g. students, part-time, self-employed). We did not control for variables associated with missing data at the 6-month and 12-month follow up or any other variable to derive trajectories as this might affect true latent class formation and classification (Asparouhov & Muthén, 2014). Parental psychological distress was included as a covariate of interest in multinomial regression analyses.

**Table S1.**

*Mean comparisons between values of responders (Not missing) and non-responders (Missing) on the SDQ scores at 6 and 12 month follow-up*

|  | 6-month | | | 12-month | | |
| --- | --- | --- | --- | --- | --- | --- |
|  | Not Missing | Missing | *p* | Not Missing | Missing | *p* |
| Reporter gender |  |  |  |  |  | 0.265 |
| Female | 1074 (34.4) | 2044 (65.6) | 0.961 | 1007 (32.3) | 2111 (67.7) |  |
| Male | 67 (34.9) | 125 (65.1) |  | 70 (36.5) | 122 (63.5) |  |
| Reporter age | 43.1 (6.0) | 42.4 (6.2) | **0.003** | 43.2 (6.0) | 42.4 (6.2) | **0.001** |
| Child Ethnicity |  |  | 0.634 |  |  |  |
| White | 1058 (34.5) | 2007 (65.5) |  | 997 (32.5) | 2068 (67.5) | 1.000 |
| BAME | 79 (32.8) | 162 (67.2) |  | 78 (32.4) | 163 (67.6) |  |
| Reporter Employment |  |  | 0.101 |  |  | **0.028** |
| Full-Time | 385 (32.1) | 813 (67.9) |  | 357 (29.8) | 841 (70.2) |  |
| Unemployed | 155 (34.6) | 293 (65.4) |  | 161 (35.9) | 287 (64.1) |  |
| Other | 603 (36.0) | 1073 (64.0) |  | 561 (33.5) | 1115 (66.5) |  |
| Income (a year) |  |  | 0.805 |  |  | 0.751 |
| Less than £16,000 | 60 (35.1) | 111 (64.9) |  | 53 (31.0) | 118 (69.0) |  |
| £16,000-£29,999 | 125 (34.7) | 235 (65.3) |  | 121 (33.6) | 239 (66.4)\| |  |
| £30,000-£59,999 | 328 (33.3) | 658 (66.7) |  | 306 (31.0) | 680 (69.0) |  |
| £60,000-£89,999 | 276 (36.8) | 475 (63.2) |  | 250 (33.3) | 501 (66.7)\| |  |
| £90,000-£119,999 | 145 (34.6) | 274 (65.4) |  | 145 (34.6) | 274 (65.4) |  |
| More than £120,000 | 140 (34.5) | 266 (65.5) |  | 127 (31.3) | 279 (68.7) |  |
| Parental distress | 4.8 (4.3) | 5.4 (4.6) | **<0.001** | 4.9 (4.2) | 5.3 (4.6) | **0.012** |

**Figure S1.**

*Non-cumulative missing data patterns across SDQ subscales for each time-point*

Note: Missing data was equal across SDQ subscales. This figure shows the number of surveys in our sample (n = 3,322) where the SDQ was not completed for a particular wave. Everyone was required to complete a survey at Time 0.

**Figure S2.**

*Non-cumulative frequency of surveys with all SDQ items completed by a parent across all time points*

Note: This figure shows the frequency of parents that completed surveys in our sample (n = 3,322). Respondents had to complete a survey at Time 0 and at least one other time point to be included in the study, thus there is no missing data on the first timepoint (Time 0). For example, 2 surveys completed (n=664) include the survey at Time 0 and one survey at any other time point.

**Appendix S2. Subgroup and Sensitivity Analysis**

**Child and Adolescent Models of Hyperactivity/Inattention Problems**

**Table S2.**

*LCGA Hyperactivity/Inattention 1-Class Model Fit Indices*

| **Model** | **AIC** | **BIC** | **RMSEA Est (90% CI)** | **CFI** | **TLI** |
| --- | --- | --- | --- | --- | --- |
| **Child Model** |  |  |  |  |  |
| Linear | 45327.05 | 45435.07 | 0.03 (0.03 -0.04) | 0.96 | 0.96 |
| Quadratic | 45289.08 | 45419.84 | 0.03 (0.03-0.04) | 0.96 | 0.96 |
| **Adolescent Model** |  |  |  |  |  |
| Linear | 21224.79 | 21320.63 | 0.03 (0.02-0.03) | 0.97 | 0.98 |
| Quadratic | 21213.08 | 21329.09 | 0.02 (0.02-0.03) | 0.98 | 0.98 |
| **Overall Model** |  |  |  |  |  |
| Linear | 66672.05 | 66788.11 | 0.03 (0.03-0.03) | 0.96 | 0.97 |
| Quadratic | 66616.78 | 66757.28 | 0.03 (0.03-0.03) | 0.97 | 0.97 |

*Note*: Estimating quadratic growth, compared to linear growth, improved the fit for the child model but not for the adolescent model (see Table 2). Therefore, we estimated linear growth for the child model and quadratic growth for the adolescent model.

**Table S3.**

*GMM Hyperactivity/Inattention 1-Class to 5-Class Model Fit Indices*

| **SDQ Subscale** | Number of Classes | AIC | BIC | Entropy Index | VLMR LRT  *p-*value |
| --- | --- | --- | --- | --- | --- |
| **Child Model (s-q@0)** |  |  |  |  |  |
|  | 2 |  |  |  |  |
|  | 3 | 45250.79 | 45398.60 | 0.55 | 0.000 |
|  | **4** | **45171.84** | **45342.40** | **0.56** | **0.001** |
|  | 5 | 45159.24 | 45352.54 | 0.56 | 0.071 |
| **Adolescent Model (s@0)** |  |  |  |  |  |
| Convergence issues | 2 | --- | --- | --- | --- |
|  | 3 | 21144.07 | 21275.22 | 0.69 | 0.574 |
|  | **4** | **21123.03** | **21274.35** | **0.76** | **0.030** |
|  | 5 | 21110.85 | 21282.35 | 0.69 | 0.323 |
| **Overall Model (s-q@0)** |  |  |  |  |  |
|  | 2 | 66717.16 | 66851.54 | 0.48 | 0.072 |
|  | 3 | 66520.38 | 66679.20 | 0.58 | 0.000 |
|  | **4** | **66412.61** | **66595.86** | **0.59** | **0.000** |
|  | 5 | 66384.44 | 66592.12 | 0.62 | 0.056 |

*Note*: Due to convergence problems we did not proceed with the 2-class model for adolescents. Random Starts were increased to XX, therefore it might be possible that we are trying to extract too many classes. The non-converged version has a higher entropy value and VLMR LRT p-value <.01.

**Income Analysis**

**Table S4.**

*Sensitivity Analysis for Income*

| Emotional | | | | | | | | | | |
| --- | --- | --- | --- | --- | --- | --- | --- | --- | --- | --- |
| Low Stable (62.2%) | High to Mod (13.6%) | | V High Stable (6.4%) | | Low to High (7.3%) | | Low-High-Low (10.5%) | | | |
| Income (>£16k) | OR (95% CI) | p | OR (95% CI) | p | OR (95% CI) | p | OR (95% CI) | | | p |
| Missing | 0.55 (0.34 – 0.89) | **0.015** | 0.53 (0.31 – 0.88) | **0.015** | 0.73 (0.36 – 1.47) | 0.374 | 0.69 (0.35 – 1.34) | | | 0.268 |
| 25% | 0.81 (0.56 – 1.17) | 0.258 | 0.71 (0.48 – 1.05) | 0.086 | 0.92 (0.55 – 1.53) | 0.745 | 0.90 (0.54 – 1.49) | | | 0.671 |
| 50% | 0.85 (0.57 – 1.28) | 0.446 | 0.69 (0.45 – 1.06) | 0.093 | 0.97 (0.55 – 1.70) | 0.907 | 0.80 (0.47 – 1.35) | | | 0.399 |
| 75% | 0.62 (0.40 – 0.95) | **0.027** | 0.53 (0.34 – 0.84) | **0.007** | 0.74 (0.41 – 1.35) | 0.326 | 0.77 (0.42 – 1.40) | | | 0.389 |
| 100% High | 0.55 (0.34 – 0.89) | **0.015** | 0.52 (0.31 – 0.88) | **0.014** | 0.74 (0.37 – 1.50) | 0.402 | 0.65 (0.34 – 1.27) | | | 0.211 |
| 100% Low | 0.82 (0.58 – 1.16) | 0.260 | 0.74 (0.51 – 1.07) | 0.112 | 0.98 (0.61 – 1.59) | 0.945 | 1.11 (0.67 – 1.83) | | | 0.688 |
| Conduct | | | | | | | | | | |
| Low Stable (84.1%) | High to Mod (4.3%) | | High Stable (8.8%) | | Very High to High (2.8%) | | --- | | | |
| Missing | 0.64 (0.34 – 1.22) | 0.177 | 0.91 (0.52 – 1.61) | 0.757 | 0.68 (0.33 – 1.38) | 0.288 | --- | | --- | |
| 25% | 0.89 (0.51 – 1.54) | 0.673 | 0.95 (0.62 – 1.45) | 0.809 | 0.73  (0.41 – 1.31) | 0.296 | --- | | --- | |
| 50% | 0.70 (0.40 – 1.22) | 0.205 | 0.91 (0.58 – 1.44) | 0.685 | 0.65 (0.36 – 1.20) | 0.173 | --- | | --- | |
| 75% | 0.72 (0.39 – 1.31) | 0.284 | 0.90 (0.54 – 1.48) | 0.667 | 0.57 (0.30 – 1.06) | 0.076 | --- | | --- | |
| 100% High | 0.64 (0.34 – 1.22) | 0.177 | 0.91 (0.52 – 1.61) | 0.757 | 0.68 (0.33 – 1.38) | 0.288 | --- | | --- | |
| 100% Low | 0.86 (0.51 – 1.44) | 0.565 | 1.03 (0.68 – 1.56) | 0.872 | 0.66 (0.38 – 1.13) | 0.131 | --- | | --- | |
| Hyperactivity/Inattention | | | | | | | | | | |
| Low Stable (64.2%) | Low to Moderate (4.8%) | | Moderate to Low (2.9%) | | Moderate Stable (28.0%) | |  | | | |
| Missing | 0.75 (0.35 – 1.59) | 0.449 | 0.75 (0.28 – 2.04) | 0.571 | 0.74 (0.51 – 1.08) | 0.121 | --- | --- | | |
| 25% | 0.86 (0.49 – 1.50) | 0.587 | 1.25 (0.53 – 2.97) | 0.611 | 0.87 (0.66 – 1.16) | 0.345 | --- | --- | | |
| 50% | 1.11 (0.57 – 2.16) | 0.753 | 1.14 (0.46 – 2.83) | 0.774 | 0.87 (0.64 – 1.17) | 0.360 | --- | --- | | |
| 75% | 0.99 (0.49 – 2.02) | 0.981 | 1.08 (0.40 – 2.91) | 0.887 | 0.82 (0.59 – 1.15) | 0.252 | --- | --- | | |
| 100% High | 0.78 (0.37 – 1.67) | 0.520 | 0.71 (0.26 – 1.92) | 0.495 | 0.74 (0.51 – 1.08) | 0.118 | --- | --- | | |
| 100% Low | 0.97 (0.56 – 1.66) | 0.898 | 1.54 (0.65 – 3.66) | 0.329 | 0.90 (0.69 – 1.17) | 0.432 | --- | --- | | |

*Note*: This table shows odds ratio and confidence intervals. Analysis were done by randomly inputting in excel all missing, 25-75% low income and 100% low or high income for individuals who selected the “prefer not to say” option in the survey.

**Appendix S3: LGCA and GMM Models Indices**

**Table S5.**

*Model Fit Indices 1-class LGCA*

| **Model** | **AIC** | **BIC** | **RMSEA Est (90% CI)** | **CFI** | **TLI** |
| --- | --- | --- | --- | --- | --- |
| **Emotion** |  |  |  |  |  |
| Linear | 79297.51 | 79413.57 | 0.04 (0.04-0.04) | 0.96 | 0.97 |
| Quadratic | 79070.44 | 79210.93 | 0.03 (0.03-0.04) | 0.98 | 0.98 |
| **Conduct** |  |  |  |  |  |
| Linear | 54024.66 | 54140.72 | 0.02 (0.02-0.02) | 0.99 | 0.99 |
| Quadratic | 53968.47 | 54108.96 | 0.02 (0.01-0.02) | 0.99 | 0.99 |
| **Hyper/Inattention** |  |  |  |  |  |
| Linear | 66672.05 | 66788.11 | 0.03 (0.03-0.03) | 0.96 | 0.97 |
| Quadratic | 66616.78 | 66757.28 | 0.03 (0.03-0.03) | 0.97 | 0.97 |

**Table S6.**

*Results for LGCA models (all within-class variances fixed to 0)*

| SDQ Subscale | Number of  Classes | AIC | BIC | Entropy Index | VLMR/LRT  p-value |
| --- | --- | --- | --- | --- | --- |
| **Emotion** |  |  |  |  |  |
|  | 2 | 87049.48 | 87177.75 | 0.90 | 0.000 |
|  | 3 | 82066.31 | 82219.02 | 0.87 | 0.000 |
|  | 4 | 80236.88 | 80414.03 | 0.83 | 0.000 |
|  | 5 | 79539.23 | 79740.80 | 0.79 | 0.026 |
|  | 6 | 79142.32 | 79368.33 | 0.77 | 0.321 |
| **Conduct** |  |  |  |  |  |
|  | 2 | 57513.39 | 57641.67 | 0.92 | 0.000 |
|  | 3 | 54809.06 | 54961.77 | 0.85 | 0.000 |
|  | 4 | 54043.50 | 54220.64 | 0.78 | 0.122 |
| **Hyperactivity/Inattention** | | |  |  |  |
|  | 2 | 69259.75 | 69388.02 | 0.78 | 0.000 |
|  | 3 | 67626.11 | 67778.82 | 0.71 | 0.000 |
|  | 4 | 67104.66 | 67281.80 | 0.64 | 0.000 |
|  | 5 | 66894.15 | 67095.73 | 0.66 | 0.000 |
|  | 6 | 66753.47 | 66979.47 | 0.63 | 0.311 |

**Table S7.**

*Results for GMM (within-class variances for slope and quadratic growth factors fixed to 0, intercept freely estimated)*

| SDQ Subscale | Number of Classes | AIC | BIC | Entropy Index | | VLMR/LRT  *p-*value |
| --- | --- | --- | --- | --- | --- | --- |
| **Emotion** |  |  |  | |  |  |
|  | 2 | 79301.58 | 79435.96 | | 0.59 | 0.000 |
|  | 3 | 78774.49 | 78933.31 | | 0.72 | 0.004 |
|  | 4 | 78383.84 | 78567.09 | | 0.71 | 0.000 |
|  | **5** | **78189.27** | **78396.96** | | **0.71** | **0.038** |
|  | 6 | 78073.85 | 78305.96 | | 0.68 | 0.072 |
| **Conduct** |  |  |  | |  |  |
|  | 2 | 53416.18 | 53550.56 | | 0.92 | 0.000 |
|  | 3 | 53157.42 | 53316.23 | | 0.91 | 0.164 |
|  | **4** | **52926.46** | **53109.71** | | **0.86** | **0.039** |
|  | 5 | 52812.11 | 53019.79 | | 0.84 | 0.335 |
| **Hyperactivity/Inattention** |  |  |  | |  |  |
|  | 2 | 66717.16 | 66851.54 | | 0.48 | 0.072 |
|  | 3 | 66520.38 | 66679.20 | | 0.58 | 0.000 |
|  | **4** | **66412.61** | **66595.86** | | **0.59** | **0.000** |
|  | 5 | 66384.44 | 66592.12 | | 0.62 | 0.056 |

**Appendix S4: Descriptive Results from Overall Mental Health Trends**

We used single growth curves to examine the changes in children and adolescent’s mental health over one year of the Pandemic. Across all time points, changes in average scores over time for emotional, conduct and hyperactivity/inattention difficulties fell within the low to moderate range. Based on graphical representation (Figure 1 of the main text), hyperactivity/inattention increased from Time 0 to May 2020 during the first national lockdown, decreased over the summer months as restrictions eased and returned to Time 0 levels in August. They decreased in September and then steadily increased from September to February as restrictions increased and the UK entered a second national lockdown, reaching their highest threshold in February, before declining in March, April and May of 2021 as the lockdown eased. In comparison, emotional difficulties remained steady across the first few months, declined around August and September when restrictions eased over the summer, and then steadily increased from September to February as the UK entered the second national lockdown, reaching their highest threshold in February, before declining around March, April, and May of 2021 back to Time 0 levels. Overall conduct difficulties followed a more stable pattern across the pandemic, with a slight increase from May to June of 2020 as restrictions increased and a slight decrease from June to December as restrictions eased. Conduct difficulties increased slightly from December to February 2021 during the second national lockdown and decreased slightly around March, April and May of 2021 as schools opened and restrictions eased.

Most difficulties increased and peaked after the national lockdowns were announced and saw a decline over periods when restrictions eased, and schools re-opened. Notably, emotional and hyperactivity/inattentive difficulties increased whilst conduct difficulties decreased slightly in October 2020 when new restrictions were announced, suggesting that spending more time in a home environment, and/or limiting social interactions reduced the likelihood of young people misbehaving at school or getting into trouble with others.

**Appendix S5: Multinomial Regression Analysis**

**Table S8.**

*Emotion – Low Stable Reference*

| Covariate  Low Stable (62.2%) | High to Mod (13.6%) | | V High Stable (6.4%) | | Low to High (7.3%) | | Low-High-Low (10.5%) | |
| --- | --- | --- | --- | --- | --- | --- | --- | --- |
|  | OR (95% CI) | p | OR (95% CI) | p | OR (95% CI) | p | OR (95% CI) | p |
| Age Group (children) | 1.27  (0.96 – 1.68) | 0.088 | 0.86  (0.63 – 1.18) | 0.351 | 1.26  (0.88 – 1.80) | 0.210 | 1.42  (0.98 – 2.05) | 0.065 |
| Child Gender (Male) | 0.63 (0.50 – 0.81) | **<0.001** | 0.39 (0.29 – 0.53) | **<0.001** | 0.66 (0.49 – 0.91) | **0.010** | 0.76 (0.55 – 1.04) | 0.082 |
| Child Ethnicity (White) | 1.50 (0.88 – 2.54) | 0.133 | 0.90 (0.53 – 1.54) | 0.703 | 1.05 (0.57 – 1.90) | 0.884 | 0.70 (0.41 – 1.19) | 0.184 |
| Income (>£16k) | 0.55 (0.34 – 0.89) | **0.015** | 0.53 (0.31 – 0.88) | **0.015** | 0.73 (0.36 – 1.47) | 0.374 | 0.69 (0.35 – 1.34) | 0.268 |
| Chronic Illness (Yes) | 1.85 (1.25 – 2.72) | **0.002** | 2.36 (1.55 – 3.59) | **<0.001** | 0.59 (0.28 – 1.27) | 0.177 | 1.17 (0.65 – 2.09) | 0.602 |
| Mental Health Condition (Yes) | 5.50 (3.23 – 9.35) | **<0.001** | 9.84 (5.85 – 16.55) | **<0.001** | 1.85 (0.73 – 4.64) | 0.193 | 2.95 (1.33 – 6.55) | **0.008** |
| SEN/ND (Yes) | 2.54 (1.86 – 3.47) | **<0.001** | 6.13 (4.42 – 8.50) | **<0.001** | 1.39 (0.87 – 2.21) | 0.173 | 1.62 (1.03 – 2.54) | **0.036** |
| DASS_9item | 1.14 (1.11 – 1.18) | **<0.001** | 1.21 (1.17 – 1.25) | **<0.001** | 1.09 (1.05 – 1.13) | **<0.001** | 1.14 (1.10 – 1.17) | **<0.001** |
| Family Conflict (Yes) | 1.55 (1.29 – 1.87) | **<0.001** | 1.63 (1.32 – 2.01) | **<0.001** | 1.30 (1.02 – 1.67) | **0.037** | 1.42 (1.11 – 1.82) | **0.005** |
| Family Warmth (Yes) | 1.02 (0.82 – 1.28) | 0.844 | 1.07 (0.83 – 1.37) | 0.600 | 0.81 (0.61 – 1.07) | 0.136 | 1.22 (0.89 – 1.69) | 0.220 |
| Friendship Quality and Support (Yes) | 0.47 (0.37 – 0.61) | **<0.001** | 0.25 (0.18 – 0.35) | **<0.001** | 0.67 (0.49 – 0.93) | **0.017** | 0.75 (0.54 – 1.04) | 0.086 |

**Table S9.**

*Emotion – High Stable Reference*

| Covariate  High Stable (10.5%) | High to Mod (13.6%) | | Low to High (6.4%) | | Low-High-Low (7.3%) | | Low Stable (62.2%) | |
| --- | --- | --- | --- | --- | --- | --- | --- | --- |
|  | OR (95% CI) | p | OR (95% CI) | p | OR (95% CI) | p | OR (95% CI) | p |
| Age Group (children) | 1.48 (1.04 – 2.12) | **0.031** | 1.46 (0.94 – 2.28) | 0.095 | 1.65 (1.05 – 2.58) | **0.030** | 1.16 (0.85 – 1.60) | 0.351 |
| Child Gender (Male) | 1.62 (1.16 – 2.25) | **0.004** | 1.69 (1.13 – 2.53) | **0.010** | 1.93 (1.29 – 2.87) | **0.001** | 2.55 (1.89 – 3.43) | **<0.001** |
| Child Ethnicity (White) | 1.66 (0.87 – 3.17) | 0.123 | 1.16 (0.55 – 2.44) | 0.696 | 0.78 (0.39 – 1.53) | 0.463 | 1.11 (0.65 – 1.89) | 0.703 |
| Income (>£16k) | 1.05 (0.63 – 1.75) | 0.861 | 1.38 (0.63 – 2.99) | 0.418 | 1.30 (0.63 – 2.69) | 0.477 | 1.90 (1.13 – 3.18) | **0.015** |
| Chronic Illness (Yes) | 0.78 (0.51 – 1.21) | 0.271 | 0.25 (0.11 – 0.57) | **0.001** | 0.49 (0.26 – 0.94) | **0.031** | 0.42 (0.28 – 0.64) | <**0.001** |
| Mental Health Condition (Yes) | 0.56 (0.35 – 0.89) | **0.013** | 0.19 (0.08 – 0.46) | **<0.001** | 0.30 (0.14 – 0.65) | **0.002** | 0.10 (0.06 – 0.17) | **<0.001** |
| SEN/ND (Yes) | 0.41 (0.29 – 0.59) | **<0.001** | 0.23 (0.14 – 0.38) | **<0.001** | 0.26 (0.16 – 0.43) | **<0.001** | 0.16 (0.12 – 0.23) | **<0.001** |
| DASS_9item | 0.95 (0.92 – 0.97) | **<0.001** | 0.90 (0.87 – 0.94) | **<0.001** | 0.94 (0.90 – 0.97) | **0.001** | 0.83 (0.80 – 0.85) | **<0.001** |
| Family Conflict (Yes) | 0.95 (0.76 – 1.20) | 0.674 | 0.80 (0.59 – 1.07) | 0.133 | 0.87 (0.65 – 1.17) | 0.357 | 0.61 (0.50 – 0.76) | **<0.001** |
| Family Warmth (Yes) | 0.96 (0.73 – 1.25) | 0.749 | 0.76 (0.54 – 1.06) | 0.106 | 1.15 (0.79 – 1.65) | 0.469 | 0.94 (0.73 – 1.20) | 0.600 |
| Friendship Quality and Support (Yes) | 1.86 (1.29 – 2.68) | **0.001** | 2.64 (1.71 – 4.06) | **<0.001** | 2.95 (1.92 – 4.52) | **<0.001** | 3.92 (2.83 – 5.43) | **<0.001** |

**Table S10.**

*Conduct –Low Stable Reference*

| Covariate  Low Stable (84.1%) | High to Mod (4.3%) | | High Stable (8.8%) | | Very High to High (2.8%) | |
| --- | --- | --- | --- | --- | --- | --- |
|  | OR (95% CI) | p | OR (95% CI) | p | OR (95% CI) | p |
| Age Group (children) | 1.17 (0.75 – 1.82) | 0.483 | 1.40 (1.00 – 1.96) | **0.050** | 1.53 (0.87 – 2.68) | 0.138 |
| Child Gender (Male) | 1.12 (0.75 – 1.68) | 0.576 | 0.98 (0.73 – 1.30) | 0.866 | 1.57 (0.93 – 2.65) | 0.094 |
| Child Ethnicity (White) | 1.82 (0.72 – 4.56) | 0.205 | 1.22 (0.69 – 2.18) | 0.494 | 1.05 (0.42 – 2.62) | 0.911 |
| Income (>£16k) | 0.70 (0.36 – 1.35) | 0.286 | 1.05 (0.58 – 1.87) | 0.880 | 0.64 (0.30 – 1.34) | 0.236 |
| Chronic Illness (Yes) | 1.51 (0.85 – 2.66) | 0.159 | 1.31 (0.83 – 2.06) | 0.250 | 1.23 (0.60 – 2.49) | 0.574 |
| Mental Health Condition (Yes) | 1.87 (1.01 – 3.45) | **0.046** | 1.16 (0.66 – 2.05) | 0.608 | 2.00 (1.00 – 3.99) | **0.050** |
| SEN/ND (Yes) | 2.99 (1.90 – 4.71) | **<0.001** | 1.83 (1.28 – 2.59) | **0.001** | 4.39 (2.55 – 7.56) | **<0.001** |
| DASS_9item | 1.04 (1.00 – 1.08) | 0.077 | 1.06 (1.03 – 1.09) | **<0.001** | 1.09 (1.04 – 1.14) | **<0.001** |
| Family Conflict (Yes) | 2.06 (1.55 – 2.75) | **<0.001** | 2.37 (1.92 – 2.93) | **<0.001** | 3.88 (2.82 – 5.35) | **<0.001** |
| Family Warmth (Yes) | 0.56 (0.41 – 0.75) | **<0.001** | 0.76 (0.60 – 0.96) | **0.024** | 0.59 (0.41 – 0.84) | **0.003** |
| Friendship Quality and Support (Yes) | 0.72 (0.46 – 1.11) | 0.138 | 0.48 (0.35 – 0.67) | **<0.001** | 0.36 (0.19 – 0.69) | **0.002** |

**Table S11.**

*Conduct – High Stable Reference*

| Covariate  High Stable (8.7%) | High to Mod (4.4%) | | Low Stable (84.1%) | | Very High to High (2.8%) | |
| --- | --- | --- | --- | --- | --- | --- |
|  | OR (95% CI) | p | OR (95% CI) | p | OR (95% CI) | p |
| Age Group (children) | 0.84 (0.50 – 1.41) | 0.501 | 0.71 (0.51 – 1.00) | **0.050** | 1.09 (0.59 – 2.02) | 0.782 |
| Child Gender (Male) | 1.15 (0.72 – 1.84) | 0.560 | 1.03 (0.77 – 1.37) | 0.866 | 1.61 (0.91 – 2.83) | 0.102 |
| Child Ethnicity (White) | 1.48 (0.52 – 4.20) | 0.457 | 0.82 (0.46 – 1.46) | 0.494 | 0.86 (0.32 – 2.34) | 0.769 |
| Income (>£16k) | 0.67 (0.30 – 1.50) | 0.329 | 0.96 (0.54 – 1.71) | 0.880 | 0.61 (0.26 – 1.44) | 0.258 |
| Chronic Illness (Yes) | 1.15 (0.59 – 2.26) | 0.678 | 0.77 (0.49 – 1.21) | 0.250 | 0.94 (0.43 – 2.03) | 0.871 |
| Mental Health Condition (Yes) | 1.61 (0.76 – 3.42) | 0.215 | 0.86 (0.49 – 1.52) | 0.608 | 1.72 (0.78 – 3.80) | 0.178 |
| SEN/ND (Yes) | 1.64 (0.96 – 2.80) | 0.070 | 0.55 (0.39 – 0.78) | **0.001** | 2.40 (1.32 – 4.38) | **0.004** |
| DASS_9item | 0.98 (0.94 – 1.03) | 0.373 | 0.94 (0.92 – 0.97) | **<0.001** | 1.03 (0.98 – 1.08) | 0.310 |
| Family Conflict (Yes) | 0.87 (0.63 – 1.21) | 0.407 | 0.42 (0.34 – 0.52) | **<0.001** | 1.64 (1.16 – 2.31) | **0.005** |
| Family Warmth (Yes) | 0.73 (0.51 – 1.04) | 0.081 | 1.32 (1.04 – 1.67) | **0.024** | 0.78 (0.53 – 1.14) | 0.201 |
| Friendship Quality and Support (Yes) | 1.48 (0.88 – 2.49) | 0.137 | 2.06 (1.50 – 2.83) | **<0.001** | 0.75 (0.38 – 1.49) | 0.411 |

**Table S12.**

*Hyperactivity/Inattention Low Stable Reference*

| Covariate  Low Stable (64.2%) | Low to Moderate (4.8%) | | Moderate to Low (2.9%) | | Moderate Stable (28.0%) | |
| --- | --- | --- | --- | --- | --- | --- |
|  | OR (95% CI) | p | OR (95% CI) | p | OR (95% CI) | p |
| Age Group (children) | 2.76 (1.75 – 4.36) | **<0.001** | 3.19 (1.74 – 5.83) | **<0.001** | 2.80 (2.25 – 3.48) | **<0.001** |
| Child Gender (Male) | 1.27 (0.89 – 1.82) | 0.193 | 1.45 (0.91 – 2.31) | 0.114 | 1.72 (1.43 – 2.06) | **<0.001** |
| Child Ethnicity (White) | 0.86 (0.45 – 1.64) | 0.642 | 1.45 (0.52 – 4.00) | 0.478 | 0.87 (0.62 – 1.22) | 0.414 |
| Income (>£16k) | 0.75 (0.35 – 1.59) | 0.449 | 0.75 (0.28 – 2.04) | 0.571 | 0.74 (0.51 – 1.08) | 0.121 |
| Chronic Illness (Yes) | 1.00 (0.51 – 1.95) | 0.999 | 1.10 (0.47 – 2.58) | 0.825 | 1.29 (0.95 – 1.75) | 0.107 |
| Mental Health Condition (Yes) | 0.85 (0.34 – 2.10) | 0.717 | 0.41 (0.07 – 2.34) | 0.317 | 1.09 (0.74 – 1.62) | 0.657 |
| SEN/ND (Yes) | 2.34 (1.43 – 3.82) | **0.001** | 1.23 (0.57 – 2.65) | 0.594 | 3.45 (2.71 – 4.39) | **<0.001** |
| DASS_9item | 1.03 (0.99 – 1.08) | 0.118 | 1.07 (1.02 – 1.13) | **0.009** | 1.10 (1.08 – 1.12) | **<0.001** |
| Family Conflict (Yes) | 1.00 (0.75 – 1.34) | 0.995 | 1.05 (0.72 – 1.53) | 0.803 | 1.29 (1.12 – 1.49) | **<0.001** |
| Family Warmth (Yes) | 1.02 (0.71 – 1.47) | 0.902 | 0.63 (0.42 – 0.95) | **0.027** | 1.05 (0.88 – 1.25) | 0.611 |
| Friendship Quality and Support (Yes) | 0.86 (0.59 – 1.25) | 0.435 | 1.28 (0.78 – 2.08) | 0.323 | 0.79 (0.66 – 0.95) | **0.014** |

**Appendix S6: Code Syntax**

**Mplus LGCA**

TITLE: emotion 1 class LGCA

DATA: FILE IS df_final_mplus.txt;

VARIABLE:

NAMES ARE ID emo_0 hyper_0 con_0

emo_1 hyper_1 con_1

emo_2 hyper_2 con_2

emo_3 hyper_3 con_3

emo_4 hyper_4 con_4

emo_5 hyper_5 con_5

emo_6 hyper_6 con_6

emo_7 hyper_7 con_7

emo_8 hyper_8 con_8

emo_9 hyper_9 con_9

emo_10 hyper_10 con_10

emo_11 hyper_11 con_11

emo_12 hyper_12 con_12

emo_13 hyper_13 con_13;

USEVAR ARE emo_0 emo_1 emo_2 emo_3

emo_4 emo_5 emo_6 emo_7 emo_8 emo_9

emo_10 emo_11 emo_12 emo_13;

IDVARIABLE IS ID;

MISSING ARE ALL (9999);

ANALYSIS:

Estimator = MLR;

MODEL:

i s q| emo_0@0 emo_1@1 emo_2@2 emo_3@3 emo_4@4

emo_5@5 emo_6@6 emo_7@7 emo_8@8 emo_9@9

emo_10@10 emo_11@11 emo_12@12 emo_13@13;

OUTPUT: SAMPSTAT; STDYX; CInterval

!to plot

PLOT: TYPE = PLOT3;

SERIES = emo_0 - emo_13 (s);

**Syntax Mplus GMM (within-class variances fixed to 0)**

TITLE: emotion 4 class sq@0 FINAL

DATA: FILE IS df_final_mplus.txt;

VARIABLE:

NAMES ARE ID emo_0 hyper_0 con_0

emo_1 hyper_1 con_1

emo_2 hyper_2 con_2

emo_3 hyper_3 con_3

emo_4 hyper_4 con_4

emo_5 hyper_5 con_5

emo_6 hyper_6 con_6

emo_7 hyper_7 con_7

emo_8 hyper_8 con_8

emo_9 hyper_9 con_9

emo_10 hyper_10 con_10

emo_11 hyper_11 con_11

emo_12 hyper_12 con_12

emo_13 hyper_13 con_13;

USEVAR ARE emo_0 emo_1 emo_2 emo_3

emo_4 emo_5 emo_6 emo_7 emo_8 emo_9

emo_10 emo_11 emo_12 emo_13;

IDVARIABLE IS ID;

CLASSES = c(4);

MISSING ARE ALL (9999);

ANALYSIS:

Estimator = MLR;

Type = MIXTURE;

STARTS = 200 50;

STITERATIONS = 20;

MODEL:

%OVERALL%

i s q | emo_0@0 emo_1@1 emo_2@2 emo_3@3 emo_4@4

emo_5@5 emo_6@6 emo_7@7 emo_8@8 emo_9@9

emo_10@10 emo_11@11 emo_12@12 emo_13@13;

s-q@0;

OUTPUT: TECH11; TECH14

PLOT: TYPE = PLOT3;

SERIES = emo_0 - emo_13 (s);

SAVEDATA:

FILE IS 'EMO_4_Final_sq.txt';

SAVE IS CPROB;

FORMAT IS FREE

**Syntax R Multivariate Analysis (Emotion example)**

setwd("~/Desktop/MplusAnalysis/FINAL/Dataset4covariates")

#install("readr")

library(readr)

cospaceT <- read_csv("EMO_5.csv")

library(dplyr)

cospaceT <- cospaceT %>% dplyr::na_if("*")

cospaceT

## Probability

probability <- cospaceT[,c("CPROB1", "CPROB2", "CPROB3", "CPROB4", “CPROB5”)] # select the probability columns

probability$max<-apply(probability, 1, max) # obtain the max value across the 4 columns

# Add the 'max' column into the data as "weight"

cospaceT$weight <- probability$max

## Create class column to text:

cospaceT$Class <- ifelse(cospaceT$C==2,"Reference", ifelse(cospaceT$C==1,"Mod2low", ifelse(cospaceT$C==3,"low2high", ifelse(cospaceT$C==4, “lowHighLow”, "highSt"))))

cospaceT$Class <- as.factor(cospaceT$Class)

#NOTE:to change the reference group change the code here.

install("mlogit")

library(mlogit)

##change variables to factors

#change to factors

cospaceT$child_gender2 <- (ifelse(cospaceT$child_gender2 == 0,"M","F"))

cospaceT$child_gender2 <- as.factor(cospaceT$child_gender2)

cospaceT$income2 <- (ifelse(cospaceT$income2 == 0,"<£16k",">£16k"))

cospaceT$income2 <- as.factor(cospaceT$income2)

cospaceT$child_ethnicity2 <- (ifelse(cospaceT$child_ethnicity2 == 0,"White","Other"))

cospaceT$child_ethnicity2 <- as.factor(cospaceT$child_ethnicity2)

cospaceT$age_group2 <- (ifelse(cospaceT$age_group2 == 0,"C","A"))

cospaceT$age_group2 <- as.factor(cospaceT$age_group2)

cospaceT$child_MH2 <- (ifelse(cospaceT$child_MH2 == 0,"No","Yes"))

cospaceT$child_MH2 <- as.factor(cospaceT$child_MH2)

cospaceT$SENND2 <- (ifelse(cospaceT$SENND2 == 0,"No","Yes"))

cospaceT$SENND2 <- as.factor(cospaceT$SENND2)

cospaceT$child_health_vulnerable <- (ifelse(cospaceT$child_health_vulnerable == 0,"No","Yes"))

cospaceT$child_health_vulnerable <- as.factor(cospaceT$child_health_vulnerable)

cospaceT$onefriend <- (ifelse(cospaceT$onefriend == 0,"No","Yes"))

cospaceT$onefriend <- as.factor(cospaceT$onefriend)

## Long data format

long_cospaceT = mlogit.data(cospaceT, choice = "Class", shape="wide")

# Model:

model_EMO <- mlogit(Class ~ 1 | age_group2 + child_gender2 + child_ethnicity2 + income2 + child_health_vulnerable + child_MH2 + SENND2 + dass_9item + parentchild_conflict + parentchild_warmth + onefriend, weights = weight, data=long_cospaceT, reflevel = "Reference")

#summary

summary(model_EMO)

#for odds ratios and confidence intervals

oddsEMO <- exp(cbind(coefficients(model_EMO), confint(model_EMO))) ## risk ratio

oddsEMO
